# Sulfur Homeostasis in Plants

**DOI:** 10.3390/ijms21238926

**Published:** 2020-11-25

**Authors:** Qian Li, Yan Gao, An Yang

**Affiliations:** State Key Laboratory of Vegetation and Environmental Change, Institute of Botany, The Chinese Academy of Sciences, Beijing 100093, China; liqian18@nwafu.edu.cn (Q.L.); duolungaoyan@foxmail.com (Y.G.)

**Keywords:** S homeostasis, S absorption, assimilation and metabolism, regulatory mechanisms

## Abstract

Sulfur (S) is an essential macronutrient for plant growth and development. S is majorly absorbed as sulfate from soil, and is then translocated to plastids in leaves, where it is assimilated into organic products. Cysteine (Cys) is the first organic product generated from S, and it is used as a precursor to synthesize many S-containing metabolites with important biological functions, such as glutathione (GSH) and methionine (Met). The reduction of sulfate takes place in a two-step reaction involving a variety of enzymes. Sulfate transporters (SULTRs) are responsible for the absorption of SO_4_^2−^ from the soil and the transport of SO_4_^2−^ in plants. There are 12–16 members in the S transporter family, which is divided into five categories based on coding sequence homology and biochemical functions. When exposed to S deficiency, plants will alter a series of morphological and physiological processes. Adaptive strategies, including *cis*-acting elements, transcription factors, non-coding microRNAs, and phytohormones, have evolved in plants to respond to S deficiency. In addition, there is crosstalk between S and other nutrients in plants. In this review, we summarize the recent progress in understanding the mechanisms underlying S homeostasis in plants.

## 1. Introduction

Sulfur (S) is one of the essential elements for growth and development, and is considered to be the fourth most important nutrient element after nitrogen (N), phosphorus (P), and potassium (K) in plants. S is a constituent of amino acids, chloroplasts, sulfatides, vitamins, coenzymes, and prosthetic groups (iron–S clusters, lipoic acid, thiamine, coenzyme A, etc.) [1,2,3]. Therefore, S plays an important role in photosynthesis, respiration, and the formation of cell membrane structures in plants. Because animals cannot synthesize S-containing amino acids, the circulation of S between plants and the environment is of great significance to the nutrition and health of humans and animals [1,2,4].

In recent years, with the gradual recognition of the importance of S, research on plant S nutrition has also received more and more attention. Indeed, there has been a systematic exploration of the absorption of S and the metabolic processes of S assimilation in plants [2,5,6,7]. Here, the recent progress associated with studies of S homeostasis in plants is summarized.

## 2. The Physiological Functions of S in Plants

S plays an important role in the process of plant resistance to biotic and abiotic stress [8,9]. There are many S-containing substances related to stress resistance in plants, including glutathione (GSH), S-containing proteins, phytochelatins, and glucosinolates. These S-containing compounds can enhance plant resistance under various stresses. For example, glutathione (GSH) is an important antioxidant substance. The redox system, composed of its reduced state and oxidized state, can eliminate the reactive oxygen species produced by oxidative stress, thereby improving the stress tolerance of plants [2,4,6,10,11]. Glucosinolate is an important secondary metabolite in plants, and its degradation products are important substances in plants for resistance to pests and herbivores [12,13,14].

S is closely related to crop yield and quality [11,15,16]. The content of S-containing amino acids in plants is an important index for evaluating crop quality. S deficiency reduces the proportion of S-containing amino acids in crop grains, whereas S application increases the content of S-containing proteins, thereby increasing the nutritional value of grains [16,17]. Furthermore, S supply levels affect wheat flour extraction rates, gluten quality, and baking quality [18]. S nutrition also affects the yield and quality of forage grass, and the application of S fertilizer can improve these factors, as well as the nutritional status of herbivorous livestock, increasing the yield and quality of wool and milk production and quality in dairy cows [19]. In addition, S nutrition also affects N fixation in legumes. For example, the application of S fertilizers could increase the capacity of N fixation in peas (*Pisum sativum* L.) and alfalfa (*Medicago sativa* L.) [20,21].

## 3. Response of Plants to S Deficiency

In recent years, due to the widespread application of high-purity S-free or low-S fertilizers, the control of industrial S-containing waste gas emissions, and intensive agriculture, S levels in the soil have been reduced, and the problem of soil S deficiency has gradually emerged [22].

Due to the poor mobility of S in plants, the symptoms of S deficiency first appear in the young parts of the plant [18]. Different types of plants have different symptoms of S deficiency, but they are all characterized by the reduced height and chlorosis of leaves (especially new leaves) [18]. The responses of plant root morphology to S-deficient conditions have also been studied [23,24,25]. Under S-deficient conditions, the root development in *Arabidopsis* was enhanced, leading to more lateral roots and higher root hair density [26]. Dan et al. found that S deficiency had little effect on primary root elongation in *Arabidopsis* [27]. However, lateral root development in *Arabidopsis* was significantly inhibited, including a decrease in the number of lateral roots, as well as the density of lateral root primordia and lateral roots. In addition, S application can increase the root length and root surface area of alfalfa grown in soil with low available S [28].

Many studies have been carried out on the response of plants to S nutrition at the physiological level [29,30,31]. Since S is a component of proteins, chloroplasts, and some important enzymes and coenzymes, S deficiency stress decreases S content and S-containing amino acids, leading to reduced metabolic activity in plants [29,30,31]. S deficiency leads to a hindrance of the synthesis of key enzymes in the process of carbon © metabolism, slows the rate of photosynthesis, and results in accumulation of more reactive oxygen species in plants [32].

Plants might reuse S nutrients under S-deficient conditions. Oilseed rape (*Brassica napus* L.) can transfer S from leaves to the root system during short-term S deficiency, thereby enhancing the recycling of S [33]. In *Medicago truncatula* plants, the distribution of photosynthetic products can be adjusted to avoid the impact of S deficiency on the next generation [34].

## 4. S Absorption in Plants

Although plant leaves can absorb gaseous SO_2_ and H_2_S, the main source of available S for plants is sulfate (SO_4_^2−^) in the soil [35]. Plant roots actively absorb SO_4_^2−^ and transport it to the aboveground parts via the xylem. Most SO_4_^2−^ is assimilated into reduced organic S in plastids (mainly in chloroplasts), while the excess SO_4_^2−^ is transported to vacuoles for storage [2,10,36]. Plant sulfate transporters (SULTRs) are responsible for the absorption of SO_4_^2−^ from the soil and the transport of SO_4_^2−^ in plants [37,38].

The plant S transporter family generally includes 12–16 genes, which are divided into five categories according to their coding sequence homology, biochemical properties, and physiological functions [39]. Mutant analysis proved that the two high-affinity S transporters, SULTR1.1 and SULTR1.2, in *Arabidopsis* are mainly located in the root hairs, root epidermis, and cortex, and they are upregulated by S deficiency. Therefore, they are considered to be mainly responsible for the absorption of S from soil [40]. Another high-affinity S transporter, SULTR1.3 in *Arabidopsis*, is located in the phloem, and is responsible for the transport of S between source and sink tissues, with its expression in roots and leaves being enhanced under S-deficient conditions [41]. In addition, the high-affinity S transporter *HVST1*, cloned in barley, has been shown to be expressed in the pericycle and xylem parenchyma cells, and may be involved in S transport in vascular tissues [42].

The second category of S transporters is mainly distributed in the xylem and phloem, and is responsible for the transport of SO_4_^2−^ between tissues. *SULTR2.1*, encoding a low-affinity S transporter in *Arabidopsis*, is expressed in the xylem parenchyma and pericycle cells, and is upregulated under S-deficient conditions [43]. SULTR2.1 is mainly responsible for the transport of S from the root system to the shoot. SULTR2.1 may mediate SO_4_^2−^ movement into the xylem parenchyma cells, and then increase the concentration of S in the symplast, thereby facilitating the unloading of S from the xylem [43]. SULTR2.1 has also been shown to be involved in the transfer of S to developing seeds [44]. A low-affinity transporter *SULTR2.2* is expressed in the phloem, and is responsible for the transfer of SO_4_^2−^ into the companion cell, which may control the S concentration in the phloem sap [45].

At present, little is known about the function of the third category of S transporter. *Arabidopsis* SULTR3.5 has been shown to assist SULTR2.1 during the transport of SO_4_^2−^ from roots to the shoots, but it cannot function as an S transporter by itself [46]. Unlike in *Arabidopsis*, SULTR3.5 can function as an S transporter in yeast [46].

SULTR4.1 and SULTR4.2 in *Arabidopsis* belong to the fourth category of S transporters. They are located in the vacuole membrane and are responsible for the transfer of SO_4_^2−^ from the vacuole. Under S-deficient conditions, the expression of *SULTR4.1* and *SULTR4.2* is upregulated, and the SO_4_^2−^ stored in the vacuole is transferred out to meet the plant’s demand for S [47]. In addition, the S transporter SULTR5.2 has been defined as a member of category 5, and is quite different from other members of the S transporter family. Nevertheless, Tomatsu et al. indicated that SULTR5.2 had been identified as a molybdenum transporter [48]. Finally, the important process of SO_4_^2−^ transportation to its assimilation site (i.e., plastids) has not yet been determined [49].

## 5. S Assimilation and Metabolism in Plants

**Figure 1:**
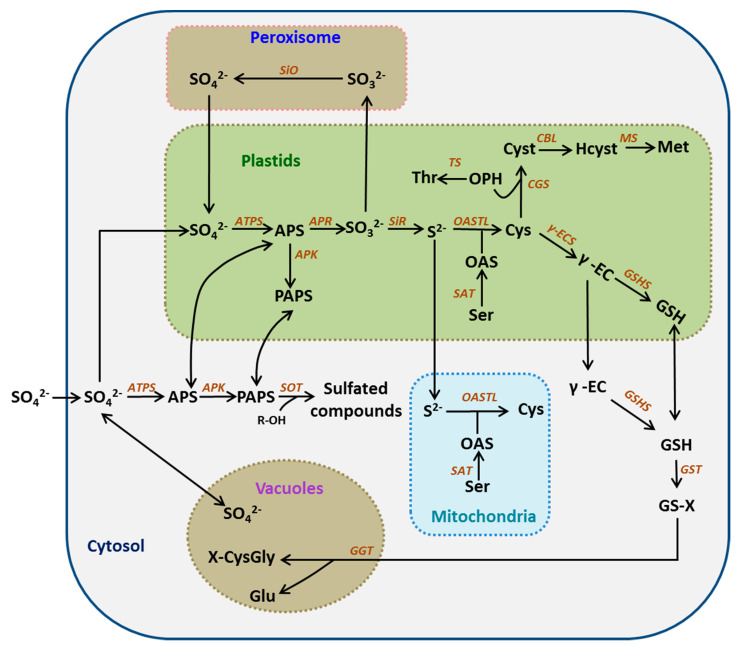
The sulfate assimilation and metabolism pathway. Enzymes are indicated in orange letters. Abbreviations of metabolites: APS, adenosine 5′-phosphosulfate; PAPS, 3′-phosphoadenosine 5′-phosphosulfate; R-OH, hydroxylated precursor; Ser, serine; OAS, *O*-acetylserine; Cys, cysteine; OPH, *O*-phosphohomoserine; Thr, threonine; Cyst, cystathionine; Hcyst, homocysteine; Met, methionine; γ-EC, γ-glutamylcysteine; GSH, glutathione; GS-X, glutathione conjugate; Glu, glutamate; X-CysGly, cysteinylglycine conjugate. Abbreviations of enzymes: ATPS, ATP sufurylase; APK, APS kinase; SOT, sulfotransferase; APR, APS reductase; SiO, sulphite oxidase; SiR, sulfite reductase; SAT, serine acetyltransferase; OAS-TL, OAS(thiol)lyase; CGS, cystathionine γ-synthase; TS, threonine synthase; CBL, cystathionine β-lyase; MS, methionine synthase; γ-ECS, γ-glutamylcysteine synthetase; GSHS, glutathione synthetase; GST, glutathione-S-transferase; GGT, γ-glutamyltransferase.

Sulfate cannot be directly used by plants. It needs to be activated into adenosine 5′-phosphosulfate (APS) and 3′-phosphoadenosine-5′-phosphosulfate (PAPS) [2,50]. ATP Sylase (ATPS) catalyzes SO_4_^2−^ to APS, with APS then phosphorylated to the storage form PAPS by APS kinase (APK). The reduction of sulfate takes place exclusively in the plastid in a two-step reaction. First, adenosine 5′-phosphosulfate reductase (APR) catalyzes the formation of sulfite from APS. Then, the sulfite reductase (SIR) catalyzes sulfite to divalent sulfide (S^2−^). Under the action of serine acetyltransferases (SAT) and O-acetylserine(thiol)lyase (OASTL), sulfide is integrated into the C scheleton of O-acetylserine (OAS), leading to the formation of the first important S-containing organic compound cysteine (Cys) [10]. More specifically, SAT catalyzes the acetylation reaction of serine and acetyl-CoA to generate OAS, while OASTL catalyzes OAS and S^2−^ to generate Cys. It is generally believed that OASTL is not the rate-limiting step in the synthesis of Cys. As a limiting factor for the generation of OAS, SAT may indirectly affect the synthesis of Cys (Figure 1) [1,2,10].

The synthesis of Cys is a key link in the process of S assimilation and metabolism, and is also the main coordination link between S, C, and N nutrition. Unlike sulfate, which is specifically reduced in plastids, Cys synthesis can be performed in plastids, mitochondria, and the cytoplasmic matrix. Cys is used as a precursor to synthesize many S-containing metabolites with important biological functions in plants [2,10,51,52,53].

### 5.1. Glutathione

The content of GSH is highest among the sulfhydryl compounds in plants. It participates in the regulation of redox and plays a role in plant resistance to adversity stress [54,55]. It is also the main form of storage and transportation of organic S in plants. Plants use Cys as a synthetic precursor to synthesize GSH through a two-step reaction. First, γ-glutamylcysteine (γ-EC) can be generated from Cys and glutamate under the action of γ-glutamylcysteine synthetase (γ-ECS), which is the rate-limiting step for the synthesis of GSH. Secondly, γ-EC interacts with glycine (or alanine) to produce GSH under the action of glutathione synthetase (GSHS) [56,57]. GSH plays a variety of important roles in maintaining the normal metabolism of plants, including being an important antioxidant, removing active oxygen and heterologous harmful substances, and protecting sulfhydryl groups in proteins and enzyme molecules [2,4,6,10,11]. GSH is also the synthetic precursor of phytochelatins, which play an important role in the process of plant resistance to heavy metal stress [58].

### 5.2. Methionine

Met is another S-containing amino acid with important biological functions, and it plays a linking role in the process of primary and secondary metabolism in plants [59,60]. Met is synthesized by a multi-step enzymatic reaction, using Cys as the synthetic precursor. Met is synthesized to S-adenosyl methionine (SAM) under the catalysis of S-adenosylmethionine synthase (SAMS), and SAM is the precursor for the synthesis of ethylene, polyamines, vitamins, coenzymes, nicotianamine (NA), and mugineic acid (MA) [6,59]. Among these derivatives, ethylene is an important hormone in plants, and NA and MA play a key role in the absorption and transport of iron and other metal ions in plants [6,59].

### 5.3. PAPS

PAPS is the storage form of APS and serves as a substrate for cytoplasmic sulfation pathways [53]. As the activated form of sulfate, PAPS participates in the synthesis and modification of many metabolites with important biological functions [4].

### 5.4. Glucosinolate

Glucosinolates are important, S-containing, secondary metabolites that are mostly found in cruciferous plants [12,13,61]. Their synthesis requires the participation of Cys, PAPS, and sulfotransferase (SOT) [62]. Glucosinolates themselves do not have physiological activity. When plants are damaged, glucosinolates are hydrolyzed by myrosinase in the cells to produce nitriles, thiocyanates, and isothiocyanates with different biological activities [63,64]. These hydrolysates have different physiological functions, including the production of peculiar aroma components in cruciferous vegetables, inhibition of microbial growth, resistance to insects and herbivores, and inactivation of cancer genes [12,13,14].

## 6. Molecular Regulatory Mechanisms of S Absorption and Assimilation in Plants

Compared with research into other macroelements, research on the regulation mechanisms of S assimilation and absorption in plants is lagging behind, and is still poorly understood [65,66]. In recent years, with the development of genomic, transcriptomic, and metabolomic research, understanding of the physiological and molecular regulatory mechanisms underlying responses to S-deficiency stress in plants has been enhanced [5,6,30,65,67,68,69].

**Figure 2:**
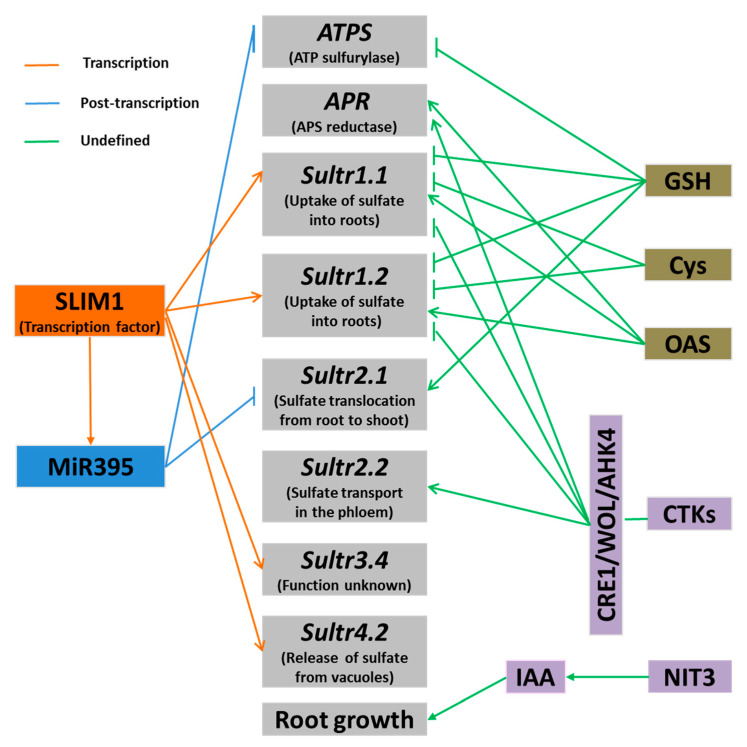
Regulatory pathways of plant’s response to sulfur deficiency. Arrows denote positive effects, whereas lines ending with a short bar indicate negative effects.

A number of studies have shown that S transporters and the S assimilation enzyme APR play a key role in S nutrition regulation [37,70]. The regulation of S absorption, transport, and assimilation in plants always follows the negative feedback mechanism driven by demand [71]. For example, the expression of genes responsible for S absorption and assimilation is upregulated under S-deficient conditions, and is reduced when S is supplied [72]. In addition, S assimilation products are involved in regulating S absorption and metabolism in plants. Cys and GSH are considered as playing a negative regulation role in S absorption and assimilation, while OAS is believed to play a positive regulation role (Figure 2) [68,73].

### 6.1. The cis-Acting Element SURE (Sulfur-Responsive Element)

Maruyama-Nakashita et al. first revealed the regulatory mechanism of S absorption and assimilation by plant at the molecular level [74]. By using various inhibitors, the regulation of *SULTR1.1* was found to occur at the transcriptional level, and required the participation of protein phosphorylase under S-deficient conditions, which suggests that the promoter of *SULTR1.1* may have a *cis*-acting element in response to S deficiency [74]. Later, a 16-base, *cis*-acting element was found in the promoter region of the S transporter *SULTR1.1* in *Arabidopsis*. The core region of this element, GAGAC, plays a key role in the response to S deficiency, and was named SURE [75]. Homologous sequences of the core region of SURE have also been found in the promoters of other S-deficiency-inducible genes, including *Arabidopsis NIT3* (NITRILASE3) and soybean seed storage protein *b*-*conglycinin*, which implies a general regulatory mechanism for S deficiency-induced gene expression [26,76]. However, the promoter region of another high-affinity S transporter *SULTR1.2* does not contain this element, indicating that the regulatory pathways of S transporters are diverse [75]. Recent studies have also shown that *SULTR1.1* is more specifically regulated by S deficiency, while *SULTR1.2* seems to be affected by the plant’s overall metabolic requirements [77]. This result also implies that SURE has specificity and limitations in regulating the response to S deficiency. In addition, the SURE element contains an auxin response factor (ARF) binding sequence (GAGACA), but SURE only plays a role in the response to S deficiency and does not respond to auxin signals [75]. Recently, the *sdi-1* gene, which is only induced by S deficiency, has been cloned in wheat. Its promoter region also contains a six-base, S deficiency response, *cis*-acting element. The position of this element in wheat *sdi-1* is the same as that of SURE in *SULTR1.1* in *Arabidopsis* [78].

### 6.2. SLIM1 Transcription Factor

SLIM1 is the first transcription factor known to be involved in the regulation of S absorption, and is responsible for the up-regulation of S transporters under S-deficient conditions [79,80]. These authors isolated the *Arabidopsis* mutant *slim1* (sulfur limitation 1) by screening mutants with a green fluorescent reporter gene. The SLIM1 gene was identified as a member of the transcription factor EIL (ETHYLENE-INSENSITIVE3-LIKE) family, with a specific role in the regulation of S nutrition. *SULTR1.2* in the *slim1* mutant cannot be upregulated by S deficiency, indicating that SLIM1 is involved in the regulation of S transporters under S-deficient conditions. Transcriptome analysis of *slim1* mutants has shown that SLIM1 regulates the S transporters *SULTR1.2*, *SULTR1.1*, *SULTR3.4*, and *SULTR4.2*, as well as a myrosinase gene. However, SLIM1 does not affect the expression of the key enzyme *APR*, which is induced by S deficiency [79]. In addition, the regulation of S transporters by SLIM1 does not require the participation of SURE elements. Therefore, the interaction between transcription factors and *cis*-acting elements in regulating the S deficiency response needs further study [79]. In addition, SLIM1 is located in the vascular tissue, but the nutrient absorption site is on the surface of the root system. Therefore, the regulation of S absorption by SLIM1 may involve long-distance signal transduction between tissues.

### 6.3. miR395

miR395 is involved in the regulation of S deficiency signals in plants, and its target genes include the low-affinity S transporter *SULTR2.1* and the three homologous genes *APS1*, *APS3*, and *APS4* encoding the APS enzyme [2,81]. S deficiency induces the up-regulation of *miR395*, and this process depends on SLIM1 [2,81]. Strangely, S deficiency enhances the expression of *miR395* and its target gene *SULTR2.1* at the same time, but tissue localization studies have shown that the expression sites of the two genes are different. miR395 is located in the phloem companion cell, and SULTR2.1 is located in the xylem parenchyma. It seems that SULTR2.1 only transports S in the xylem. Similarly, due to constraints of spatial location, the expression of *SULTR2.1* may not be fully regulated by miR395 [2,81]. In addition, miR395 is also involved in regulating the accumulation of Sin leaves and transporting it from old leaves to new leaves [81,82].

## 7. The Role of Hormones in the Regulation of S Nutrition in Plants

Phytohormones are involved in the regulation of S absorption, transport, and assimilation metabolism in plants.

### 7.1. Cytokinins

Cytokinins (CTKs) inhibit the expression of S transporters *SULTR1.1* and *SULTR1.2*, thereby negatively regulating S uptake in *Arabidopsis*. This process is dependent on cytokinin receptors CRE1/WOL/AHK4 [83]. However, CTKs do not affect the induction of S absorption under S-deficient conditions, indicating that the negative regulation of cytokinins and the signal pathway of S absorption under S-deficient conditions are two independent pathways [83]. In addition, studies have reported that exogenous treatment with cytokinin induces the expression of the S transporter *SULTR2.2* and the key S assimilation enzyme *APR* [84]. These results suggest that the role of cytokinins in regulating the plant’s response to S deficiency is not very clearly understood.

### 7.2. Auxin

Some progress has been made on the role of auxin in the regulation of root morphology under S-deficient conditions [26,85]. As mentioned earlier, glucosinolates are decomposed into SO_4_^2−^ and indole-3-acetonitrile (IAN) by myrosinase. IAN can be converted into indole acetic acid (IAA) under the action of nitrilase (NIT). There are four nitrilase genes in *Arabidopsis*, three of which (*NIT1*, *NIT2*, and *NIT3*) can convert IAN to IAA [86]. S deficiency enhances the expression of the nitrilase-encoding gene *NIT3* in *Arabidopsis*, and then promotes the conversion of glucosinolates to IAN. Therefore, it has been speculated that the synthesis of auxin is enhanced to promote root development in *Arabidopsis* under S-deficient conditions [26]. However, there is no difference in auxin content in plants growing under S-deficient and S-sufficient conditions [26]. In addition, transcriptome studies have shown that S deficiency can induce IAA synthesis-related genes, which may increase the synthesis of auxin, thereby enhancing *Arabidopsis* root development under S-deficient conditions [85]. By using the DR5::GUS reporter strain, the inhibition of lateral root development in *Arabidopsis* by S deficiency was found to be due to reduced auxin synthesis or auxin sensitivity, which indicates that auxin negatively regulates the response of *Arabidopsis* to S deficiency [27]. Therefore, auxin may regulate root morphology under S-deficient conditions through both positive and negative feedback pathways [87,88]. First, S deficiency upregulates the *NIT3* and myrosinase-related genes, thereby increasing the synthesis of auxin to enhance root growth [26,85]. On the other hand, the increased auxin contents change cell calcium ion concentrations and then upregulate the expression level of calmodulin. Calmodulin acts on the auxin-related transcription factor IAA28, which may inhibit the expression of auxin-induced genes [89]. In addition to the positive regulation pathway, in which S deficiency enhances root growth by increasing auxin synthesis, there is an IAA28-mediated negative feedback regulation pathway that prevents the enhancement of plant root development under S-deficient conditions, and in turn initiates a negative feedback regulation mechanism for restricting root growth [87,88]. Although this evidence indicates that auxin is involved in regulating the response of root morphology to S deficiency, its mechanism remains to be clarified.

Furthermore, auxin has no effect on the upregulation of high-affinity plant S transporter expression under S-deficient conditions [74]. SURE, the key regulatory element of the S deficiency response, contains a binding sequence (GAGACA) for the auxin response factor. However, there is no evidence to indicate that SURE is related to auxin signaling [75]. Recent studies have shown that the overexpression of auxin-related genes in response to S deficiency changes many metabolic processes in plants without affecting S metabolism [90].

### 7.3. Other Hormones

The ethylene signal pathway may be related to the response of tobacco LSU (response to low sulfur) family of proteins under S-deficient conditions [91,92]. Transcriptome analysis has shown that jasmonic acid (JA) is related to the expression of genes involved in S metabolism, and JA synthesis-related genes are induced by S deficiency [93,94]. These results imply that JA plays a positive role in the regulation of S metabolism. Recently, gibberellin has been implicated in the regulation of APR transcription and enzyme activity in *Arabidopsis*. JA, salicylic acid, ethylene, and CKs only regulate APR enzyme activity, whereas ABA (abscisic acid) does not affect the upregulation of APR under salt stress [95].

## 8. Systemic Signals Involved in Regulation of Plant S Nutrition

Systemic signals (i.e., long-distance signals) are messengers that connect plant roots and shoots. They can regulate the overall relationship between nutrient requirements in plants and nutrient supply in soil. Several S-deficiency-responsive genes (such as *APR3*) have been shown to be regulated by systemic signals, and are believed to be related to the decrease in OAS content in *Arabidopsis* [96]. However, systemic signals that are involved in regulation of plant S nutrition are relatively elusive [97]. In the process of regulating the expression of S transporters and S absorption activity, no systemic signals from the shoots to the roots were found in rape plants under S-deficient conditions [98]. In *Arabidopsis*, *SULTR1.1*, and *SULTR1.2* are only regulated by local S deficiency, but not affected by systemic signals. In addition, the expression of *SULTR1.1* and *SULTR1.2* does not depend on OAS or GSH [77]. Overall, there are conflicting conclusions about whether plants have a systemic response to S-deficiency stress. Specific systemic signal molecules have not been determined. Further study is needed to elucidate the regulation mechanism of systemic signals under S-deficient conditions.

## 9. Crosstalk between S and Other Nutrition in Plants

There is a tendency for the balance of nutrient metabolism to be maintained in the plant body, so the interaction between nutrient elements will affect the plant response to the stress of mineral nutrient deficiency [99]. However, there are relatively few reports on the interaction between S and other nutrients [8]. Therefore, more attention needs to be paid to the mechanism of crosstalk between S and other forms of nutrition in plants [100].

### 9.1. S and N

There is an interaction between the regulation of S and N nutrition metabolism in plants [101]. N deficiency inhibits the response of *SULTR1.1* and *SULTR1.2* to S deficiency in *Arabidopsis* [74]. N deficiency downregulates expression of the key enzyme APR during S assimilation, and the Cys precursor OAS may be a node that connects N and S metabolism [102,103]. Indeed, N supply can induce the upregulation of genes responsible for S absorption and assimilation, thereby increasing the plant’s S absorption efficiency [104].

A lack of S nutrition affects the utilization of N in plants. S deficiency inhibits the uptake of nitrate and reduces the activity of nitrate reductase, leading to the accumulation of nitrate and a reduction in N utilization in plants [105]. S-deficiency stress leads to a decrease in the content of S and S-containing amino acids in plants, which may be the cause of hindered protein synthesis and the accumulation of non-protein forms of N [106]. However, in a study of rapeseed plants under short-term S deficiency, the total N content and N absorption were not affected [33].

### 9.2. S and C

The absorption and metabolism of S are also affected by C metabolism in plants [101]. Sugar can upregulate the expression of the S transporters *SULTR1.1* and *SULTR1.2*, and increase the activity and transcription level of APR [107,108]. Under CO_2_-free conditions, the absorption and transport of S are inhibited, while the activity and transcription level of APR are decreased in plants [107].

### 9.3. S and P

There is a correlation between S and P nutrition metabolism in plants [109]. Sulfolipids are quickly synthesized to replace phospholipids under P-deficient conditions, and phospholipids can also replace sulfolipids under S-deficient conditions [110]. S and P transporters have similar topological structures and molecular regulation mechanisms [111]. For example, the absorption and transport of S and P in plants are both negatively regulated by cytokinins [83,112]. *miR395*, which is involved in the regulation of the S deficiency response [81], is also upregulated under P-deficient conditions [113]. Transcriptome analysis has shown that P deficiency upregulates the expression of S transporters in *Arabidopsis* [114]. PHR1 (Phosphate Response 1), which is a key transcription factor in the P-deficiency signaling pathway, may play an important role in the P and S nutrient interaction in *Arabidopsis* [115]. *SULTR1.3*, which is responsible for the transport of S in shoots, is upregulated by P deficiency, and its promoter region contains a PHR1 binding sequence [115]. By using *phr* mutants, the regulation of *SULTR1.3* by P deficiency was proven to depend on PHR1 [115]. PHR1 also inhibits the expression of other S transporters (*SULTR2.1* and *SULTR3.4*) [115]. Furthermore, the PHR1 homologous gene *PSR1* in *Chlamydomonas reinhardtii* also negatively regulates S transporters [116]. Nevertheless, P deficiency has not shown any effect on the expression of *SULTR1.1* and *SULTR1.2* in *Arabidopsis* [74].

Overall, the regulation of a plant’s response to S deficiency is interrelated with the metabolism of N, C, and P; however, the specific regulation mechanisms are still not very well known.

## 10. Conclusions

S nutrition plays an important role in the growth and development of plants. It is closely related to the response of plants to biotic and abiotic stresses, as well as the yield and quality of crops. Soil S deficiency has gradually become one of the main factors limiting plant growth and crop yields. However, compared with studies on other macronutrient elements, there is not enough research on plant S nutrition, and a mechanistic understanding of the absorption, metabolism, and regulation of S nutrition remains elusive. Therefore, more effort is needed to understand the plant’s response to soil S deficiency stress and its regulatory mechanisms, and this will form the basis for improving S nutrient utilization efficiency in crops.
